# Genoprotective potential of *Macaranga* species phytochemical compounds on HT-29 human colorectal adenocarcinoma cell line

**DOI:** 10.1186/s41021-023-00282-5

**Published:** 2023-10-30

**Authors:** Ee Ling Siew, Lishantini Pearanpan, Zhafri Zamkhuri, Fariza Juliana Nordin, Theng Choon Ooi, Kok Meng Chan, Aisyah Salihah Kamarozaman, Norizan Ahmat, Nor Fadilah Rajab

**Affiliations:** 1 ASASIpintar Program, Pusat PERMATA@Pintar Negara, Universiti Kebangsaan Malaysia, Bangi, Selangor 43600 Malaysia; 2 Faculty of Health Sciences, Universiti Kebangsaan Malaysia, Jalan Raja Muda Abd Aziz, Kuala Lumpur, 50300 Malaysia; 3 Center for Healthy Ageing and Wellness, Faculty of Health Sciences, Universiti Kebangsaan Malaysia, Jalan Raja Muda Abd Aziz, Kuala Lumpur, 50300 Malaysia; 4 Department of Biological Sciences and Biotechnology, Faculty of Science and Technology, Universiti Kebangsaan Malaysia, Bangi, Selangor 43600 Malaysia; 5 Center for Toxicology and Health Risk Studies, Faculty of Health Sciences, Universiti Kebangsaan Malaysia, Jalan Raja Muda Abd Aziz, Kuala Lumpur, 50300 Malaysia; 6 Product Stewardship and Toxicology, Group Health, Safety and Environment (GHSE), Petroliam Nasional Berhad (PETRONAS), Kuala Lumpur, 50088 Malaysia; 7 Centre of Foundation Studies, Universiti Teknologi MARA, Cawangan Selangor, Kampus Dengkil, Dengkil, Selangor 43800 Malaysia

**Keywords:** Genoprotective, Oxidative stress, DNA damage, *Macaranga heynei*

## Abstract

**Background:**

The species of genus *Macaranga* are widely found in Malaysian secondary forests and has been used as an alternative for treating varieties of illness. Studies have shown that the medicinal properties of this genus contain anti-inflammatory, antioxidant, and anti-cancer effects. This study aimed to determine the cytotoxicity of six isolated phytochemicals from *Macaranga heynei (M. heynei), Macaranga lowii and Shorea leprosula* on HT-29 human colorectal adenocarcinoma cell lines.

**Results:**

One out of six isolated phytochemical compounds, identified as “Laevifolin A”, showed a cytotoxicity with an IC_50_ value of 21.2 µM following 48 h treatment as detected using Sulforhodamine B (SRB) assay. Additionally, no induction of apoptosis and oxidative stress were observed on Laevifolin A treated HT-29 cells as determined using Annexin V-FITC/PI assay and dihydroethidine (HE) staining, respectively. Additionally, no damage to the DNA were observed as measured using the Alkaline Comet assay. Further investigation on menadione-induced oxidative DNA damage showed the genoprotective potential of Laevifolin A on HT-29 cells.

**Conclusions:**

In conclusion, this study indicated that only one compound (Laevifolin A) that extracted from *M. heynei* has the cytotoxicity potential to be developed as an anticancer agent in human colorectal adenocarcinoma. However, besides exhibiting cytotoxic effect, the compound also exhibits genoprotective capability that warrant further investigation.

## Introduction

Cancer is one of the leading causes of death globally, and despite in the advances in drug development, it is of great importance to developing new plant-derived medicines. Changes in dietary consumption among the Asian populations leads to the increased incidence of colorectal cancer in Malaysia [1]. Furthermore, the end-stage (Stage IV) of colorectal cancer such as adenocarcinoma was reported to be the highest among the cases of colorectal cancer [2]. Colorectal cancer is highly lethal and very malignant due to the difficulty in early diagnosis, highly metastasis, invasive and poor prognosis [3, 4].

Therefore, the search for a new alternative chemotherapeutic drug has caught the attention of scientists for ages. Many studies have been focusing on the development of natural compounds as agent for cancer therapies which derived from plants or animals [5, 6]. Furthermore, many phytochemicals have been suggested as anticancer adjuvant therapies because of their anti-proliferative and pro-apoptotic properties [7, 8]. Therefore, the continuing search for plant-based anticancer agents is essential to discover new therapeutic agent for colorectal cancer treatment [9, 10].

*Macaranga* is a genus under the family of Euphorbiaceae and can be found in the secondary forest of Peninsular Malaysia, Sumatra, and Borneo [11]. Phytochemical studies of *Macaranga* species have shown that this genus has abundant sources of flavonoids particularly prenylated flavonoids and stilbenoids [12, 13]. *Macaranga heynei (M. heynei)* or locally known in Peninsular Malaysia and Thailand as ‘Mahang Biru’ contained various phytochemicals that may exert anticancer activities [14]. In the previous study, six different compounds were successfully isolated and characterized from *M. heynei*, *Macaranga lowii* (*M. lowii*) and *Shorea leprosula* (*S. leprosula*), namely Laevifolin A, Laevifolin B, Malayheyneiin A, Laevifonol, Hopeaphenol and Pentadecyl ferulate which were further used in this study [14].

DNA damage occurs both endogenously and exogenously by producing insults such as free radicals, and topological changes, each causing distinct forms of measureable damage [15]. DNA damage itself will cause cell cycle arrest where it leads to abnormal repair of the DNA or cell death via apoptosis [16]. Therefore, in this study, the cytotoxicity and mode of cell death of *M. heynei, M. lowii and S. leprosula* compounds were conducted using HT-29 human colorectal adenocarcinoma cell line. Further investigation was carried out to assess the genoprotective potential of the isolated compounds using HT-29 cells treated with menadione, a redox cycling agent. Most importantly, this provides a better understanding of the properties of this medicinal plant for further development of novel anticancer agent.

## Materials and methods

### Reagents

Phosphate Buffered saline (PBS), trypsin-EDTA solution, sulforhodamine B (SRB) salt, hydroethidine (HE), hydrogen peroxide and menadione were purchased from Sigma-Aldrich, St. Louis, MO, USA. The cell culture medium McCoy’s 5a Modified Medium, Eagle’s Minimum Essential Medium (EMEM), foetal bovine serum (FBS), penicillin-streptomycin antibiotic, non-essential amino acids (NEAA) were from Thermo Fisher Scientific, Waltham, MA, USA. Annexin assay kit containing buffer solution, Annexin V-FITC and Propidium iodide solution were from BD Biosciences, San Jose, CA, USA. All reagents used in the present study were of analytical grade purity and cell culture use.

### Plant material

*M. heynei, M. lowii and S. leprosula* were collected from Perak, Selangor and Pahang respectively and identified by a botanist, Dr. Shamsul Khamis. The voucher specimen SK2875/15 (*M. heynei*) and SK1634/09 (*S. leprosula*) were deposited at Herbarium of Universiti Putra Malaysia while FSG8 (*M. lowii*) was deposited at the herbarium of Forest Research Institute Malaysia (FRIM).

### Test compounds

**Fig. 1:**
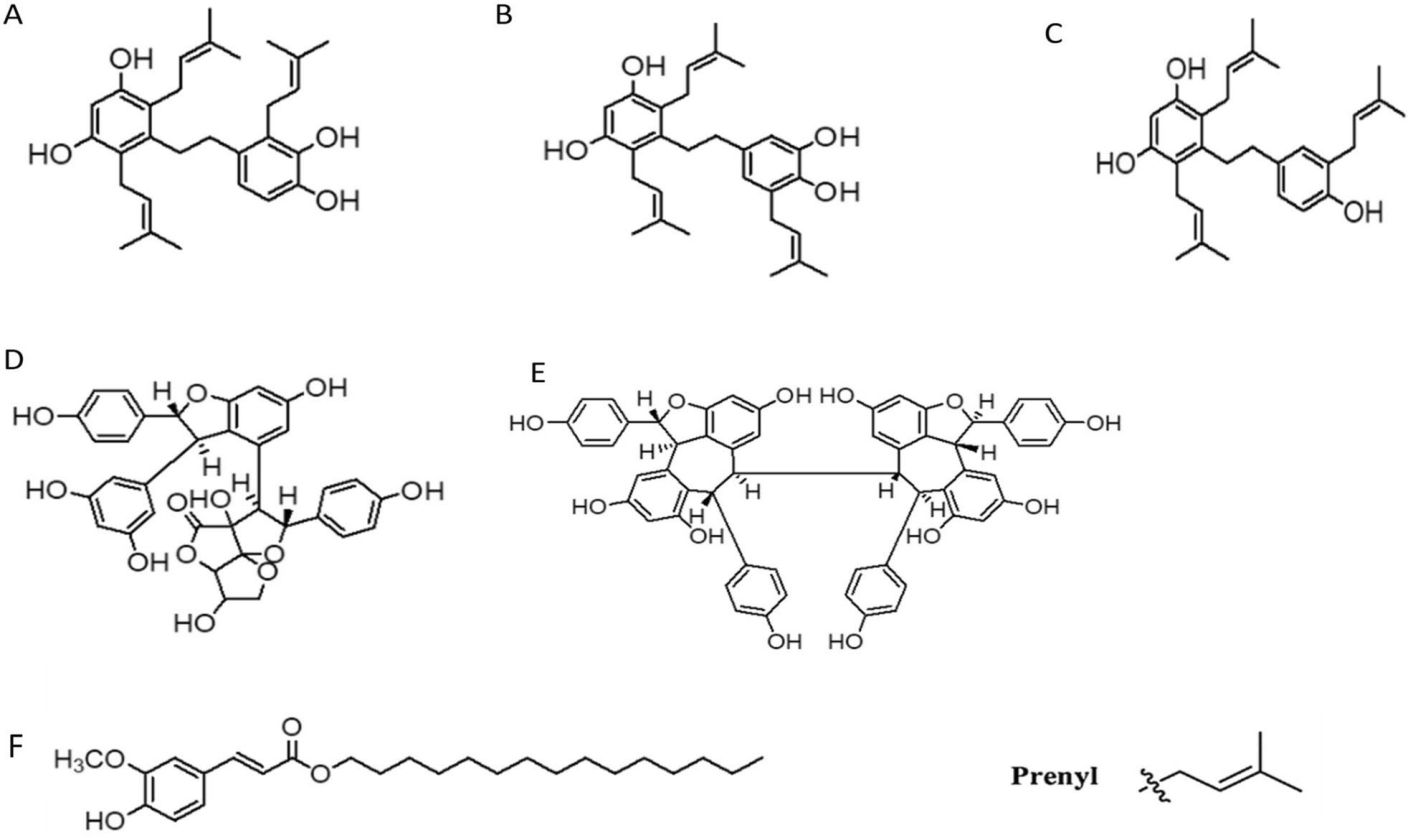
Structures of compounds isolated from *M. heynei, M. lowii and S. leprosula*. **a** Laevifolin A, **b**) Laevifolin B, **c**) Malayheyneiin A, **d**) Laevifonol, **e**) Hopeaphenol and **f**) Pentadecyl ferulate. A prenyl group is a 5-carbon structure known as 3-methylbut-2-en-1-yl

Test compounds were isolated from *M. heynei, M. lowii and S. leprosula* and provided by Dr. Aisyah Salihah Kamarozaman from the Universiti Teknologi Mara (UiTM). All compounds, namely Laevifolin A (MW = 450.594), Laevifolin B (MW = 450.594), Malayheyneiin A (MW = 434.594), Laevifonol (MW = 628.564), Hopeaphenol (MW = 906.896) and Pentadecyl ferulate (MW = 404.57) were dissolved in dimethyl sulfoxide (DMSO; Thermo Fisher Scientific, Waltham, MA, USA) as a 6 mg/mL stock solution. The structures of all compounds are depicted in Fig. 1.

### Cell culture

HT-29 human colorectal adenocarcinoma cell line (with epithelial morphology that was isolated from colorectal adenocarcinoma) and CCD-18Co human normal colon cell line (exhibiting fibroblast morphology that was isolated from the normal colon tissue) were obtained from the American Type Culture Collection (ATCC; Manassas, VA). HT-29 (HTB-38™) cells were grown in McCoy’s 5a Modified Medium (Sigma-Aldrich, St. Louis, MO, USA) while CCD-18Co (CRL-1459™) cells were grown in Eagle’s Minimum Essential Medium (EMEM; Thermo Fisher Scientific, Waltham, MA, USA). Both culture media were supplemented with 10% foetal bovine serum (FBS; Thermo Fisher Scientific, Waltham, MA, USA) and penicillin-streptomycin antibiotic (Thermo Fisher Scientific, Waltham, MA, USA). Non-essential amino acids (NEAA; Thermo Fisher Scientific, Waltham, MA, USA) were added to the completed EMEM media. Cells were maintained at 37 °C in a 5% CO_2_ incubator. Cells were detached via trypsinization (0.025% trypsin) after reaching confluency and were ready to be used for tests.

### Cytotoxicity testing

Sulforhodamine B (SRB) assay was used to assess the cytotoxicity of the compounds [17]. Briefly, HT-29 and CCD-18Co cells were seeded in 96-well-plate at 1.5 × 10^5^ cells/ml respectively and were incubated at 37 °C in 5% CO_2_ for 24 h. 100 µl of six concentrations of serially diluted compounds (0.94–30 µg/ml) were added in their respective wells. Hydrogen peroxide (10 mM) was used as the positive control. The plate was incubated for 48 h. Cells were fixed with 50 µl of 50% cold (4 °C) trichloroacetic Acid (TCA) and incubated at 4 °C for 1 h. The plates were washed five times with tap water and air-dried before staining with 100 µL of 0.4% (w/v) SRB staining solution (Sigma-Aldrich, St. Louis, MO, USA). Further incubation was done for 30 min at room temperature. Subsequently, the plates were washed three times with 1% (v/v) acetic acid to remove any unbound stains. After air-dried, 200 µL of 10 mM trizma base were added into each well and agitated for 15 min. Absorbance was read with a microplate reader with Microplate Manager® Software at the wavelength of 564 nm. All experiments were carried out in triplicates.

### Flow cytometric analysis of apoptosis by using annexin V-FITC/PI assay

The mode of cell death was determined using the Annexin V-FITC/PI apoptosis assay [18]. Cells were treated with IC_50_ value of the selected compound and apoptosis assessment were done at 4 h, 24 and 48 h respectively. 100µM of menadione was used as a positive control with an enriched media act as a negative control. The measurement of apoptosis is based on phosphatidylserine (PS) exposure as described previously. Briefly, cells were collected and resuspended in 150 ml annexin V buffer containing 2.5 ml FITC-conjugated annexin V and incubated for 15 min in the dark. Propidium iodide (10 ml of 50 mg/ml stock in PBS) was then added and samples were subjected to flow cytometric analysis using FACS CANTO II (BD Biosciences, San Jose, CA, USA).

### Flow cytometric analysis of reactive oxygen species (ROS) using dihydroethidine (HE)

The generation of superoxide anion was determined as described previously [18]. Briefly, 1 ml of 10 mM HE was added to 1 ml of HT-29 treated cells at IC_50_ value of selected compound and further incubated for 15 min at 37 ± 1 °C. Cells were then centrifuged at 220 g for 5 min and resuspended in 1 ml PBS. Flow cytometry was performed using FACS CANTO II (BD Bioscience).

### Alkaline comet assay

Alkaline Comet Assay was used to assess strand breaks in DNA indicative of DNA damage [19]. The experiment was carried out according to previous study by Tan et al. [20]. HT-29 cells were treated with the Laevifolin A at the IC_50_ value (21.2 µM) for 30 min, 1 h, 4 and 24 h. Cells were also treated with hydrogen peroxide (H_2_O_2_) (as positive control, 1.0 mM) for 30 min. Meanwhile, to investigate the protective effects of Laevifolin A against menadione-induced genotoxicity, HT-29 cells were pre-treated with Laevifolin A at its IC_50_ value for 20 h, followed by a 4-hour incubation with menadione (100 µM). Following respective incubations, detached cells in the media were collected, added to trypsinize cells and centrifuged (2500 rpm/5 min). The supernatant was removed, and the pellet was washed with Ca^2+^−/Mg^2+^-free PBS before re-centrifugation. Pellets left at the bottom were mixed thoroughly with 80 µl of 0.6% low melting agarose (LMA) (w/v) and the mixture was pipetted onto hardened 0.6% normal melting agarose (NMA) (w/v) on the slide. Coverslips were placed to spread the mixture and the slides were left on ice for LMA to solidify. Following the removal of the coverslips, the embedded cells were lysed in lysis buffer containing 2.5 M NaCl, 100 mM Na_2_EDTA, 10 mM Tris, and 1% Triton X-100 for 1 h at 4 °C. Slides were soaked in electrophoresis buffer solution for 20 min at 4 °C for DNA-unwinding before electrophoresis at 300 mA, 25 V, for 20 min. Subsequently, the slides were rinsed with neutralizing buffer for 5 min and stained with 50 ml ethidium bromide solution. Slides were analysed using Leitz Laborlux epifluorescence microscope equipped with 515 barrier filter and 560 emission filter. Fifty cells per slide were scored and the tail moment (TM) and % tail DNA (TD) were analyzed with COMET assay III (Perceptive Instruments, UK).

### Statistical analysis

The data were presented as the mean ± standard error of mean (SEM) with three independent experiments. Statistical significance between means was assessed using ANOVA followed by a Dunnet’s t-test. A *p*-value of < 0.05 was considered significant.

## Results

### Cytotoxicity

**Fig. 2:**
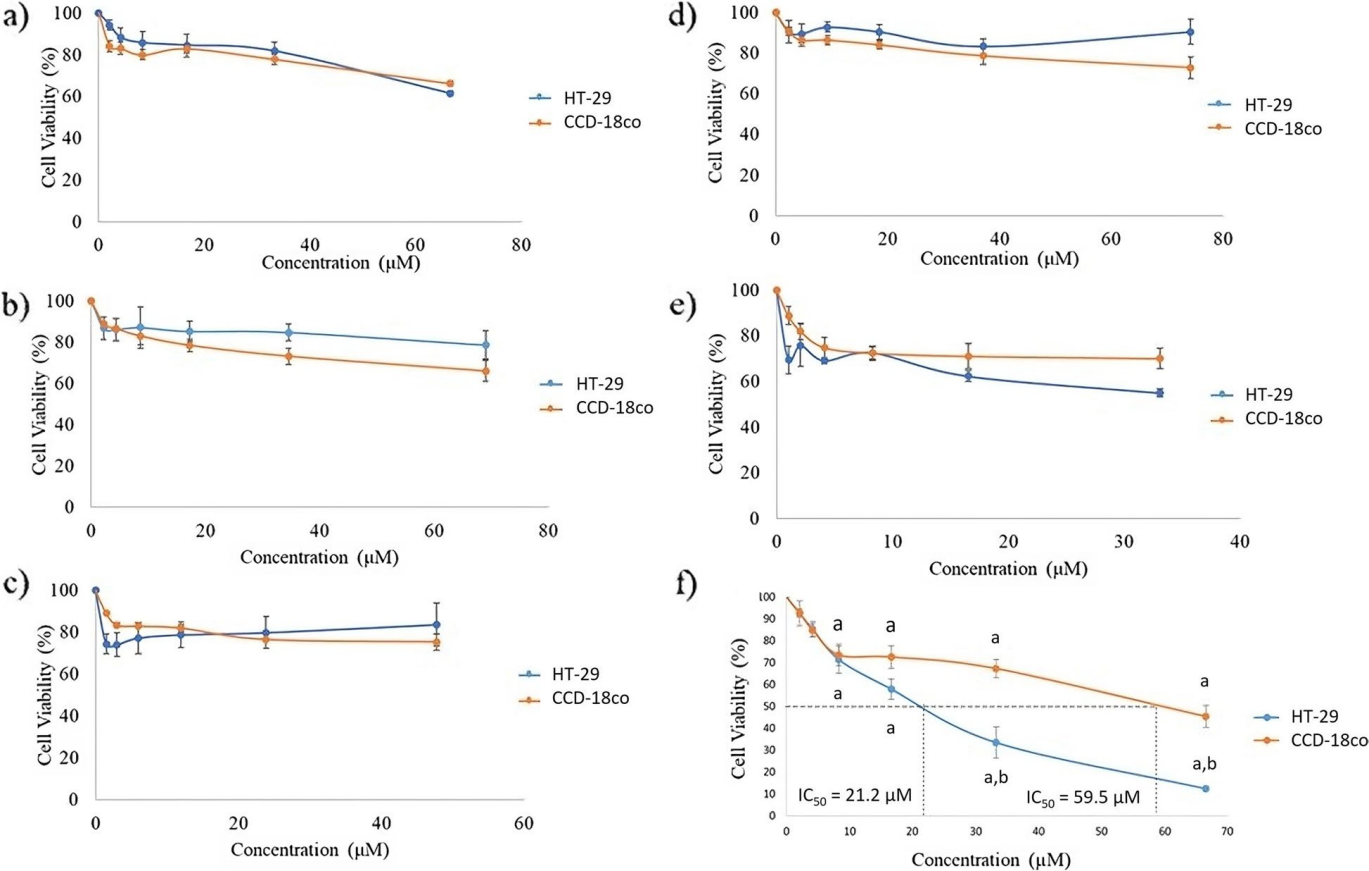
Cytotoxicity of **a**) Laevifolin B, **b**) Malayheyneiin A, **c**) Laevifonol, **d**) Pentadecyl ferulate, **e**) Hopeaphenol and **f**) Laevifolin A against HT-29 and CCD-18Co cells at 48 h. The results are expressed as mean ± SE of at least three independent experiments. ^a^ Significant difference (*p* < 0.05) as compared with negative control. ^b^ Significant difference (*p* < 0.05) as compared with CCD-18Co cells

The cytotoxic effects of the six phytochemicals were investigated using the SRB assay on HT-29 and CCD18-Co cells as shown in Fig. 2. HT-29 and CCD18-Co cells showed a significant (*p* < 0.05) decrease in viability in a concentration-dependent manner after 48 h treatment with Laevifolin A, which obtained IC_50_ values of 21.2 µM and 59.5µM respectively (Fig. 2f). Interestingly, the activity of Laevifolin A was significantly lower on CCD18-Co cells when compared to the tumor cells, suggesting a tumor specific effect with IC_50_ of 21.2 µM (*p* < 0.05). On the other hand, no IC_50_ values were observed for the other 5 phytochemicals. Therefore, for further analysis, only Laevifolin A compound was selected to determine its mode of cell death, possible generation of ROS and DNA damage induction.

### Mode of cell death

**Fig. 3:**
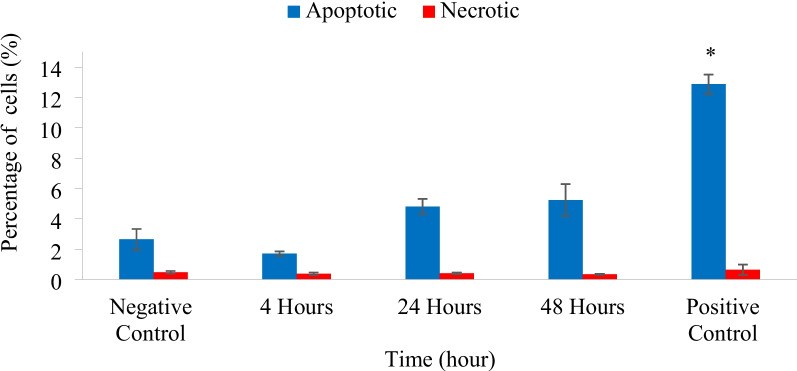
Percentage of apoptotic and necrotic HT-29 cells treated with IC_50_ value of Laevifolin A. The results are expressed as mean ± SE of at least three independent experiments. ^*^ Significant difference (*p* < 0.05) as compared with negative control

The capability of Laevifolin A (IC_50_: 21.2 µM) to induce apoptosis on HT-29 cells was investigated using Annexin V-FITC/PI assay at 4 h, 24 and 48 h as shown in Fig. 3. However, there was no significant induction of apoptotic cells (*p* > 0.05) observed as compared to negative control. This result indicates that the cytotoxic effect observed was not caused by the loss of integrity on the membrane of the cell. Menadione as a positive control at 100 µM showed a significant (*p* < 0.05) increase of apoptotic cells as anticipated.

### Generation of ROS

**Fig. 4:**
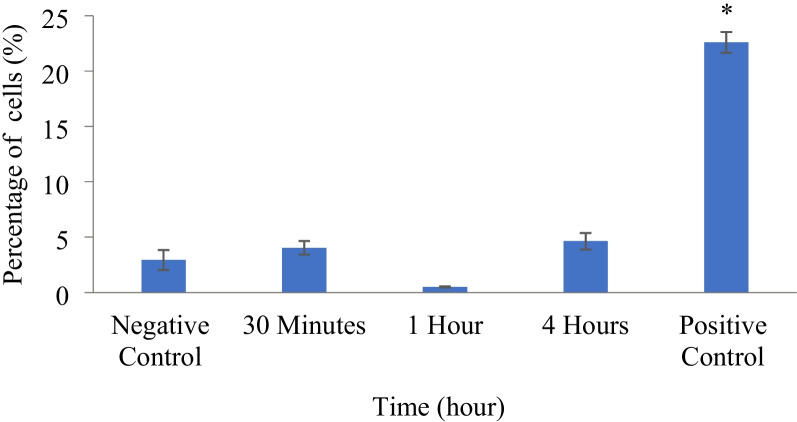
Reactive oxygen species production was measured by dihydroethidium oxidation using flow cytometry. Percentage of dihydroethidium positive HT-29 cells following treatment with IC_50_ value (21.2 µM) of Laevifolin A. The results are expressed as mean ± SE of at least three independent experiments. ^*^ Significant difference (*p* < 0.05) as compared with negative control

The generation of ROS in Laevifolin A-treated HT-29 cells was assessed using dihydroethidine. The uptake of dihydroethidium stain were indicative of the production of ROS in the cells. Our results showed no significant increase in the percentage of cells with HE uptake (*p* > 0.05) following treatment with Laevifolin A as compared with the negative control (2.9 ± 0.9%) (Fig. 4). Menadione, reactive oxygen species (ROS) generator, at 100 µM caused a significant (*p* < 0.05) increase in the generation of ROS (22.6 ± 1.0%) as anticipated.

### Genotoxicity

Alkaline Comet assay was employed to identify the potential of Laevifolin A to induce DNA damage on HT-29 cells, as shown in Table 1. DNA damage was assessed based on the TM and TD values. Our results showed no significant difference in TM and TD at all time points as compared to the negative control following treatment with the IC_50_ value of Laevifolin A. Hydrogen peroxide (1 mM) that was used as positive control detected a significant increase in DNA damage with TM value of 14.91 ± 1.5 and TD of 22.6 ± 1.0, respectively. However, as shown in Table 2, pre-treatment of Laevifolin A showed a significant (*p* < 0.05) reduction of DNA damage in TD and TM values, 1.24 ± 0.1 and 9.8 ± 0.9, respectively, when treated with menadione (100 µM). This indicated the potential protective effect of Laevifolin A against menadione, a potent oxidative DNA damaging agent.

**Table 1:** Level of DNA damage of Laevifolin A treated HT-29 cells

Treatment	Level of DNA damage (Arbitrary unit)	
	Tail moment	% tail DNA
Negative control	0.25 ± 0.1	3.5 ± 1.1
30 min	0.53 ± 0.1	6.4 ± 1.6
1 h	0.45 ± 0.03	6.0 ± 0.6
4 h	0.31 ± 0.1	4.3 ± 1.2
24 h	0.69 ± 0.1	7.8 ± 0.7
Positive control (1 mM H_2_O_2_ for 30 min)	*14.91 ± 1.5	*22.6 ± 1.0

The results are expressed as mean ± SE of three independent experiments

*p < 0.05 versus negative control

**Table 2:** Level of DNA damage of Menadione on pre-treated Laevifolin A HT-29 cells

Treatment	Level of DNA damage (Arbitrary unit)	
	Tail moment	% tail DNA
Negative control	0.26 ± 0.1	3.1 ± 0.4
Menadione	*7.00 ± 0.8	*23.4 ± 3.1
Laevifolin A + Menadione	*^,#^1.24 ± 0.1	*^,#^9.8 ± 0.9
Positive control (1 mM H_2_O_2_ for 30 min)	*^,#^20.92 ± 4.5	*^,#^39.8 ± 2.0

The results are expressed as means ± SE of three separate experiments

*p < 0.05 versus negative control

^#^*p* < 0.05 vs menadione

## Discussion

According to the American Cancer Society (2017), cancer is known to be the second most common cause of death following cardiovascular diseases. In Malaysia, colorectal cancer is the second most commonly diagnosed cancer [2]. The rise in cancer incidence along with the unwanted side effects associated with chemotherapy urged the discovery of new agents from natural resources [21]. Phytochemicals use in cancer chemoprevention has been proven effective against different malignancies [22, 23]. Secondary plant-derived metabolites are potentially an inexhaustible source of chemicals for new drug development. Phenolic compounds produced by plants as secondary metabolites consists of one or more aromatic ring that is/are attached to hydroxyl groups. These phenolic compounds have the potential to act as potent antioxidant or prooxidant effects [24]. Plant from the genus *Macaranga* is rich in phenolic compounds particularly prenylated flavonoids, stilbenoids, terpenoids as well as tannins [25, 26].

Our results demonstrated that only Laevifolin A out of the six compounds isolated from *M. heynei, M. lowii and S. leprosula* that been investigated on HT29, human colorectal carcinoma cell line, exhibited cytotoxic effect with an IC_50_ value of 21.2 µM. Indeed, Laevifolin A, isolated from *Macaranga rubiginosa*, was reported to induce cell death in murine leukemia P-388 cells, suggesting that the cytotoxic effects of Laevifolin A may not be limited to colorectal cancer cells alone [27]. On the other hand, no IC_50_ values were obtained for both Laevifolin B and Malayheyneiin A treated HT-29 cells although there is a slight drop on the cell viability. According to Kamarozaman et al. (2018), this could be due to the presence of the prenyl group (Fig. 1) at C2 of ring B, causing steric hindrance [14]. Whereby, Laevifolin B and Malayheyneiin A are less hindered, considered weak due to their loose structure, thus limit its penetration into the cells. In addition, the steric hindrance in Laevifolin A increases the accessibility to exert its effect on cells [25]. Consistent with our present results, Tanjung et al. (2017) had shown that the IC_50_ value of Laevifolin A (4.3 ± 0.6 µM) in P-388 cells was significantly lower than that of Laevifolin B (12.3 ± 0.6 µM) [27].

In this study, the assessment of mode of cell death of Laevifolin A treated HT-29 cells were determined using Annexin-V-FITC assay. Restoration of the apoptotic pathway by drugs targeting both apoptotic pathways constitute is a promising anticancer therapeutic pathway as an approach to be developed as an anticancer agent [28]. However, our data showed Laevifolin A did not induce apoptosis in HT-29 cells as mode of cell death. This result possibly indicated that the cytotoxicity that was observed is not caused by the loss of integrity of the cell membrane [29].

It is known that ROS can damage macro biomolecules such as proteins, lipids, and DNA and reduce the DNA repair capability that led to the transformation of normal cells to cancerous cells by mutating the key genes [30]. The changes of ROS from its optimum level either by increase or decrease of ROS could result in cell death. However, in this study, no significant increase or decrease of ROS were observed following treatment with Laevifolin A up to 4 h. In fact, a previous study has demonstrated that Laevifolin A possesses antioxidant properties, as confirmed by the DPPH radical scavenging activity assay [25]. This characteristic may be attributed to the presence of phenolic structures, which can function as free radical scavengers and antioxidants [24]. Furthermore, our current findings also suggest that Laevifolin A did not induce DNA damage in HT-29 cells even after treatment for up to 24 h. Given that Laevifolin A did not provoke ROS production and DNA damage in the earlier time points preceding cell death, we presumed that ROS and DNA damage are not the primary inducers of cell death in Laevifolin-treated HT-29 colorectal cancer cells. Moreover, as cancer cells typically demonstrate elevated basal levels of ROS due to imbalance between oxidants and antioxidants, it is worth noting that low to moderate levels of ROS have been reported to stimulate the proliferation, migration, and invasion of cancer cells [31]. Therefore, Laevifolin A may exert its anti-proliferative effects by reducing ROS levels in HT-29 cells, thereby suppressing the proliferation of these cancer cells. However, further studies are needed to identify the exact mechanisms underlying the cytotoxic effects of Laevifolin A against HT-29 colorectal cells.

While the antioxidant properties of Laevifolin A have been reported in the previous study, we have further investigated the genoprotective potential of Laevifolin A against menadione, the potent ROS inducer. Menadione is a vitamin K analogue also known as vitamin K3 [25, 32]. It is also a well-known compound to induce oxidative stress, inflammation, and apoptosis [33]. In vitro generation of ROS may cause modifications and damage to almost all cellular chemical components, including lipid peroxidation, as well as an aggregation and denaturation of proteins. Free radicals also induce changes in DNA leading to its mutations or cytotoxic effects [34, 35]. Accumulation of ROS causes an imbalance in redox status and produces oxidative stress that will further affects the DNA. Our data indicated that there was inhibition of oxidative DNA damage induced by menadione following pre-treatment of Laevifolin A in HT-29 cells. This finding suggested that Laevifolin A isolated from *M. heynei* has the genoprotective potential possibly via the decrease in superoxide anions generated by menadione. Furthermore, phenolic compounds are known to exert free-radical-scavenging activity induced by an oxidative damaging agent such as Menadione [36]. Thus, Laevifolin A may modulate the intracellular antioxidant responses, such as enzymatic and non-enzymatic antioxidant mechanisms, to confer protection against oxidative stress that warrant further study.

In summary, Laevifolin A exhibited cytotoxicity and genoprotective potential on menadione-induced oxidative DNA damage in HT-29 human colorectal adenocarcinoma cells. This study indicated that Laevifolin A from *M. heynei* may have great potential to be developed as a novel anticancer agent. However, further investigation is needed to better understand its possible mechanism as an anticancer agent in colorectal cancer.

## Data Availability

The datasets used and/or analyzed during the current study are available from the corresponding author on reasonable request.
